# The novel nutritional metabolic risk index and established triglyceride-glucose indices in type 2 diabetes: modifying role of biological aging assessed by telomere length

**DOI:** 10.3389/fnut.2026.1911472

**Published:** 2026-08-12

**Authors:** Chan Yang, Juan Li, Xiaowei Liu, Xiaoxia Li, Yuhong Zhang, Yi Zhao

**Affiliations:** 1 School of Nursing, Ningxia Medical University, Yinchuan, Ningxia, China; 2 Health Administration and Social Services College, Ningxia polytechnic University, Yinchuan, China; 3 School of Public Health, Ningxia Medical University, Yinchuan, Ningxia, China

**Keywords:** effect modification, geriatric nutritional risk index, telomere length, triglyceride glucose index, type 2 diabetes mellitus

## Abstract

**Background:**

Triglyceride-glucose (TyG) derivatives are recognized markers of type 2 diabetes (T2DM) risk. However, the association between composite indices integrating nutritional and metabolic status and biological aging in T2DM patients remains unclear.

**Methods:**

Data from 4,572 adults were analyzed to evaluate the association between four indices, including the geriatric nutritional risk index (GNRI), TyG, triglyceride glucose-waist-to-height ratio (TyG-WHtR), and the composite nutritional metabolic risk index (NMRI), and T2DM prevalence using logistic regression and restricted cubic spline analysis. The potential mediating and moderating roles of relative telomere length (RTL) were also explored.

**Results:**

TyG and TyG-WHtR were strongly associated with higher odds of T2DM, with TyG-WHtR presenting the strongest association (odds ratio [OR] = 10.20; 95% confidence interval [CI]: 8.64–12.10). NMRI was independently inversely associated with T2DM in a non-linear manner, and the inverse association was stronger among participants with shorter RTL (interaction OR = 1.007; 95% CI: 1.000–1.014; *p* = 0.041). RTL did not mediate but moderated the NMRI–T2DM link. GNRI exhibited no significant association with T2DM.

**Conclusion:**

TyG and TyG-WHtR may serve as useful indicators of T2DM risk, while NMRI is inversely associated with T2DM. RTL appeared to modify the association between NMRI and T2DM, suggesting potential value for individualized metabolic risk stratification, which warrants further investigation in longitudinal studies. These findings suggest that the interplay between nutritional-metabolic indices and biological aging in diabetes pathogenesis warrants further investigation.

## 1 Introduction

Type 2 diabetes mellitus (T2DM) is a major global health concern, progressing from impaired glucose tolerance to full diabetes, underscoring the need for early risk detection (1). Key factors in T2DM development include insulin resistance (IR) and chronic metabolic dysregulation, which disrupt glucose balance and damage organs (2). IR is characterized by reduced insulin sensitivity in the liver, muscle, and fat, leading to decreased glucose uptake and increased hepatic glucose production (2). Chronic metabolic issues, such as hyperglycemia, abnormal lipid levels, and obesity, exacerbate IR (3).

Given the central role of insulin resistance and metabolic dysfunction in T2DM pathogenesis, identifying practical biomarkers for early risk assessment is of considerable clinical importance. Identifying simple and reliable clinical markers is crucial for assessing IR. The triglyceride-glucose (TyG) index, calculated from fasting triglycerides (TG) and fasting plasma glucose (FPG), is widely used due to its simplicity and strong correlation with IR (4, 5). To enhance its predictive power, TyG is often combined with body composition or obesity measures, creating derivative indices (6). Among these, the TyG index with waist-to-height ratio (TyG-WHtR) is a promising indicator, reflecting both metabolic issues and central obesity. In contrast to these metabolic dysfunction markers, the geriatric nutritional risk index (GNRI) represents a distinct class of biomarkers focused on nutritional status. Based on serum albumin and body weight, was initially created to evaluate nutritional risk and predict mortality in the elderly (7). Recent studies have indicated a strong link between GNRI and metabolic health, influenced by nutritional status, affecting insulin sensitivity and inflammation (8, 9). Nutritional status significantly impacts metabolic health. Protein-energy malnutrition can cause sarcopenia and increased inflammation, while overnutrition worsens metabolic issues through adipose dysfunction and oxidative stress (8). GNRI can detect a broad spectrum of nutritional issues, ranging from malnutrition to overnutrition, highlighting its relevance to metabolic health.

The combination of metabolic dysregulation and malnutrition may heighten health risks. To address this, researchers developed the nutritional metabolic risk index (NMRI) by merging GNRI and TyG-WHtR to assess nutritional and metabolic risks (10, 11). Although previous studies have primarily evaluated NMRI as a predictor of mortality, its association with T2DM requires further validation in varied populations (6, 12). Additionally, the mechanisms linking NMRI to T2DM and its role in biological aging are not well understood (13).

Understanding biological aging is essential, as it involves cellular senescence-related processes, such as telomere attrition, mitochondrial dysfunction, oxidative stress, and chronic inflammation. Telomere length, a marker of senescence, is linked to T2DM risk (14, 15), with shortening accelerating β-cell aging and reducing insulin secretion (14, 16). Nutritional and metabolic states can affect telomere shortening through oxidative stress, inflammation, and epigenetic pathways (16–18). We explored whether biological aging may be associated with the relationship between nutritional-metabolic risk and T2DM, although this question has not been tested using indices such as GNRI or TyG.

Therefore, this study aimed to evaluate and compare the associations of four indices (GNRI, TyG, TyG-WHtR, and NMRI) with T2DM risk in a large cohort and explore whether relative telomere length (RTL) mediates or moderates these associations.

## 2 Materials and methods

### 2.1 Study design and population

**Figure 1:**
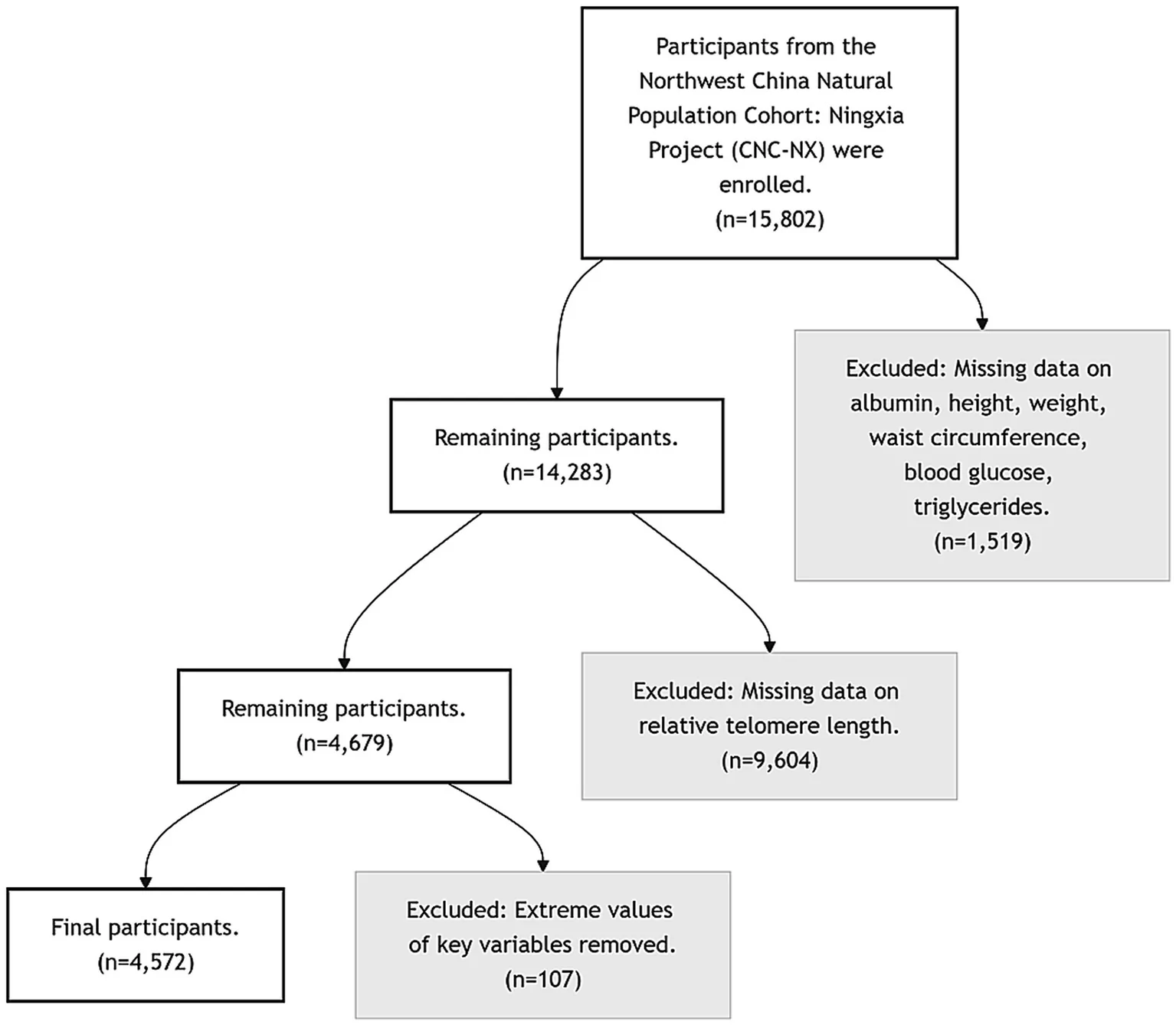
Flow diagram of study.

A cross-sectional study was conducted using data from the Northwest China Natural Population Cohort: Ningxia Project (CNC-NX), part of China’s National Key Research and Development Program. The baseline survey, conducted from June 2018 to August 2020, included questionnaire surveys, physical examinations, and biochemical analyses of blood and urine samples. A total of 15,802 participants were initially recruited. After sequential exclusion of 1,519 with missing core biochemical or anthropometric data, 9,604 with missing RTL data, and 107 with extreme key variable values, 4,572 participants aged ≥30 years with complete diabetes diagnosis and nutritional metabolic risk index data were included in the final analysis (Figure 1). Given the cross-sectional study design, all analyses in this work were exploratory rather than confirmatory.

This study was conducted in accordance with the Declaration of Helsinki and was approved by the Ethics Review Board of Ningxia Medical University (ID: 2018-012), with written informed consent obtained from all participants.

### 2.2 Data collection and laboratory measurements

Trained interviewers used standardized questionnaires to collect data on demographics, lifestyle, and disease history. Height was measured using a portable stadiometer. Waist circumference (WC) and hip circumference (HC) were measured using a standard non-elastic tape measure, and the mean of two measurements was recorded. Weight was assessed using a bioelectrical impedance analyzer (InBody 370). Blood pressure was measured using a digital sphygmomanometer (OMRON-7124) after a 5–10 min rest. Fasting blood samples were collected after an 8-h fast and processed by trained nurses according to a standardized protocol. Samples were centrifuged, aliquoted, transported under cold-chain conditions, and stored at −80 °C.

Serum levels of total cholesterol (TC), TG, high-density lipoprotein cholesterol (HDL-C), low-density lipoprotein cholesterol (LDL-C), albumin, and FPG were determined using automated biochemical analyzers (Mindray BS-430). Genomic DNA was extracted from blood samples using an Omega Bio-tek kit. RTL was quantified using real-time polymerase chain reaction (RT-PCR) (Takara kit) with the 36B4 gene as a reference. RT-PCR consisted of an initial denaturation at 95 °C for 30 s, followed by 40 cycles of denaturation at 95 °C for 5 s, and annealing at 60 °C for 30 s. A final melt curve analysis was performed to verify amplification specificity. Samples were analyzed in duplicate, and TB Green dye fluorescence was recorded. The telomere-to-single-copy gene (T/S) ratio, indicating RTL, was calculated using the ΔCt method (19).

### 2.3 Diagnostic criteria and definitions of study variables

T2DM was diagnosed according to the Chinese Guideline for the Prevention and Treatment of T2DM (2020 Edition) (20). T2DM was defined as FPG level ≥ 7.0 mmol/L, the presence of classic hyperglycemic symptoms (polyuria, polydipsia, polyphagia, and unexplained weight loss), or a self-reported physician-diagnosed diabetes.

The nutritional metabolic risk indices were calculated as follows. GNRI = 1.489 × serum albumin (g/L) + 41.7 × (actual body weight/ideal body weight). Ideal body weight was estimated using the Lorentz formula, adjusted for gender and height (21). TyG = ln [fasting TG (mg/dL) × FPG (mg/dL)/2] (22). TyG-WHtR = TyG × [WC (cm)/height (cm)]. NMRI = GNRI/TyG-WHtR (12).

The analysis included covariates such as age, gender, education, marital status, occupation, tea consumption and alcohol intake, smoking status, physical activity, medical history of hypertension, cardiovascular disease (CVD), and stroke, and family history of diabetes, CVD, and stroke. Smoking was defined as lifetime consumption of ≥ 100 cigarettes, alcohol consumption as drinking weekly in the past year, and tea consumption as having a weekly habit in the past year. Physical activity was classified as insufficient, sufficient, or high based on meeting the 150 min per week criterion. Additionally, homocysteine, TC, HDL-C, LDL-C, serum creatinine (SCr) levels were included. Hypertension was defined according to the 2018 Chinese Guidelines for the Management of Hypertension as systolic blood pressure ≥ 140 mmHg and/or diastolic blood pressure ≥ 90 mmHg (23). A positive family history of diabetes, hypertension, and CVD indicated that at least one parent had the condition. Personal histories of CVD and stroke were self-reported.

### 2.4 Statistical analysis

Statistical analyses were conducted using R software (version 4.5.0), with a two-sided *p*-value <0.05 considered significant. Missing data were addressed using multiple imputation. Continuous variables, assessed for non-normality, were summarized as medians with interquartile ranges (IQRs) and compared using the Mann–Whitney *U* test. Categorical variables were presented as frequencies (percentages) and analyzed using the chi-square test.

Logistic regression analysis was performed to evaluate associations between indices and T2DM, with results expressed as odds ratios (ORs) with 95% confidence intervals (CIs). Three models were constructed: Model 1 was unadjusted; Model 2 was adjusted for age and gender; and Model 3 was adjusted for various demographic, lifestyle, and health factors. No significant multicollinearity among covariates was detected. Additionally, quartile-based analyses were conducted to examine dose–response relationships.

To compare the predictive performance of the four indices, receiver operating characteristic (ROC) curves were constructed and the area under the curve (AUC) was calculated. The integrated discrimination improvement (IDI) and net reclassification improvement (NRI) were computed to quantify the incremental predictive value of adding each index to Model 3. The DeLong test was used to compare differences in AUCs between indices.

Participants were stratified into short RTL and long RTL groups based on the median RTL value. Separate logistic regression analyses were performed for each group to examine whether the associations between metabolic indices and T2DM risk differed by RTL status.

Restricted cubic spline (RCS) analysis with four knots was performed to examine potential nonlinear associations between continuous indices and T2DM, with nonlinearity tested using the likelihood ratio test.

Mediation analysis was performed using the ‘mediation’ package in R to assess whether RTL mediated the associations between metabolic indices and T2DM. The average causal mediation effect (ACME) and average direct effect (ADE) were estimated with 1,000 bootstrap resamples.

Moderation analysis was conducted to examine whether RTL modified the associations between indices and T2DM. Multiplicative interaction terms (NMRI × RTL group) were introduced into the regression models. Stratified analyses and visualization were performed to interpret significant interactions.

Subgroup analyses were conducted to examine the robustness of findings across different populations.

## 3 Results

### 3.1 Baseline characteristics of the study population

The final analysis included 4,572 participants, of whom 1,015 had T2DM. Supplementary Table 1 presents the baseline characteristics of both the T2DM and non-T2DM groups. Compared to the non-T2DM group, the T2DM group was older and had significantly higher values for BMI, WC, FPG, TC, TG, TyG, TyG-WHtR and NMRI (all *p* < 0.001), while GNRI values and RTL were significantly lower (*p* < 0.05).

### 3.2 Association analysis (primary association + predictive performance)

Table 1 presents the logistic regression analysis examining the associations between primary exposure indices and T2DM risk. When analyzed as continuous variables in the unadjusted model (Model 1), TyG (OR = 8.30; 95% CI: 7.17–9.65) and TyG-WHtR (OR = 8.35; 95% CI: 7.20–9.72) were positively associated with T2DM risk (all *p* < 0.001). NMRI exhibited a significant inverse association with T2DM risk (OR = 0.97; 95% CI: 0.96–0.97; *p* < 0.001). In contrast, the association between GNRI and T2DM was not statistically significant (*p* = 0.082). These associations remained statistically significant after adjustment for age and gender (Model 2). In the fully adjusted model (Model 3), TyG (OR = 10.10; 95% CI: 8.53–11.90) and TyG-WHtR (OR = 10.20; 95% CI: 8.64–12.10) remained positively associated with T2DM risk (all *p <* 0.001). The inverse association between NMRI and T2DM risk persisted (OR = 0.97; 95% CI: 0.96–0.97, *p* < 0.001), while no significant linear association was observed for GNRI (OR = 1.00; 95% CI: 0.99–1.01; *p* = 0.500).

**Table 1:** Associations between GNRI, TyG, TyG-WHtR, NMRI and T2DM based on weighted logistic regression analysis.

Characteristic		Model 1			Model 2			Model 3		
		OR	95% CI	*p*-value	OR	95% CI	*p*-value	OR	95% CI	*p*-value
GNRI		1.01	1.00, 1.02	0.082	1.00	0.99, 1.01	>0.900	1.00	0.99, 1.01	0.500
Quartile	Q1	Ref			Ref			Ref		
	Q2	1.01	0.83, 1.24	>0.900	0.97	0.79, 1.19	0.800	0.90	0.73, 1.11	0.300
	Q3	1.11	0.91, 1.35	0.300	1.01	0.83, 1.24	>0.900	0.94	0.77, 1.16	0.600
	Q4	1.20	0.99, 1.47	0.064	1.04	0.85, 1.27	0.700	0.95	0.77, 1.17	0.600
TyG		8.30	7.17, 9.65	<0.001	8.68	7.47, 10.1	<0.001	10.10	8.53, 11.90	<0.001
Quartile	Q1	Ref			Ref			Ref		
	Q2	2.79	1.97, 4.03	<0.001	2.78	1.96, 4.02	<0.001	2.88	2.02, 4.19	<0.001
	Q3	6.67	4.83, 9.42	<0.001	6.75	4.87, 9.55	<0.001	7.43	5.31, 10.60	<0.001
	Q4	29.10	21.30, 40.70	<0.001	30.50	22.30, 42.80	<0.001	35.20	25.2, 50.40	<0.001
TyG_WHtR		8.35	7.20, 9.72	<0.001	8.72	7.50, 10.20	<0.001	10.20	8.64, 12.10	<0.001
Quartile	Q1	Ref			Ref			Ref		
	Q2	2.78	1.95, 4.03	<0.001	2.76	1.93, 4.01	<0.001	2.85	1.99, 4.16	<0.001
	Q3	7.35	5.31, 10.40	<0.001	7.42	5.35, 10.5	<0.001	8.17	5.84, 11.70	<0.001
	Q4	28.80	21.00, 40.40	<0.001	30.30	22.00, 42.60	<0.001	34.80	24.80, 49.90	<0.001
NMRI		0.97	0.96, 0.97	<0.001	0.97	0.96, 0.97	<0.001	0.97	0.96, 0.97	<0.001
Quartile	Q1	Ref			Ref			Ref		
	Q2	0.25	0.21, 0.30	<0.001	0.24	0.20, 0.29	<0.001	0.23	0.19, 0.28	<0.001
	Q3	0.10	0.08, 0.13	<0.001	0.09	0.07, 0.12	<0.001	0.08	0.07, 0.11	<0.001
	Q4	0.04	0.03, 0.05	<0.001	0.03	0.03, 0.05	<0.001	0.03	0.02, 0.04	<0.001

OR, Odds Ratio; CI, Confidence Interval. Model 1: unadjusted; Model 2: adjusted for Age, Gender; Model 3: adjusted for Age, Gender, Education, Marriage, Occupation, Tea consumption status, Alcohol drinking status, Smoking status, Exercise level, History of hypertension, History of CVD, History of stroke, Family history of diabetes, Family history of CVD, Family history of stroke, HCY, TC, HDL-C, LDL-C, SCr.

Analysis based on quartile groups revealed that higher quartiles of TyG and TyG-WHtR were associated with an increased risk of T2DM, with the participants in the highest quartile (Q4) presenting significantly higher risks (OR = 35.20; 95% CI: 25.20–50.40 for TyG; OR = 34.80; 95% CI: 24.80–49.90 for TyG-WHtR) compared to the lowest quartile (Q1). In contrast, T2DM risk decreased in the NMRI Q4 group with higher quartiles, and no significant association was found between GNRI quartiles and T2DM risk.

To formally compare the predictive performance of these indices, ROC analysis was performed. As presented in Supplementary Figure 1 and Supplementary Table 2, TyG and TyG-WHtR showed the highest AUC values (0.841 and 0.837, respectively), significantly higher than NMRI (*p* < 0.001). Furthermore, NRI and IDI analyses indicated that TyG (IDI = 0.236, NRI = 0.866) and TyG-WHtR (IDI = 0.227, NRI = 0.847) provided substantial incremental predictive value beyond the Model 3, while NMRI showed moderate improvement (IDI = 0.167, NRI = 0.781). Pairwise comparisons using the DeLong test, with full results presented in Supplementary Table 3, showed that TyG and TyG-WHtR had significantly higher AUC values than NMRI (both *p* < 0.001), while NMRI significantly outperformed GNRI (*p* < 0.001). TyG also showed slightly higher AUC than TyG-WHtR (*p* = 0.003).

### 3.3 Stratified analysis by RTL

Participants were stratified into short- and long-RTL groups based on the median RTL value. Separate logistic regression analyses were conducted for each group, and the results are presented in Supplementary Tables 4, 5. In the fully adjusted model, TyG (OR = 8.73; 95% CI: 6.96–11.00 for short RTL; OR = 13.10; 95% CI: 10.20–16.90 for long RTL), TyG-WHtR (OR = 8.65; 95% CI: 6.88–11.00 for short RTL; OR = 13.80; 95% CI: 10.70–18.00 for long RTL), and NMRI (OR = 0.96; 95% CI: 0.96–0.97 for short RTL; OR = 0.97; 95% CI: 0.96–0.97 for long RTL) were significantly associated with T2DM risk in both groups (all *p* < 0.001). The associations for TyG and TyG-WHtR were stronger in the long RTL group, while the inverse association of NMRI was slightly stronger in the short RTL group.

### 3.4 Dose–response analysis

**Figure 2:**
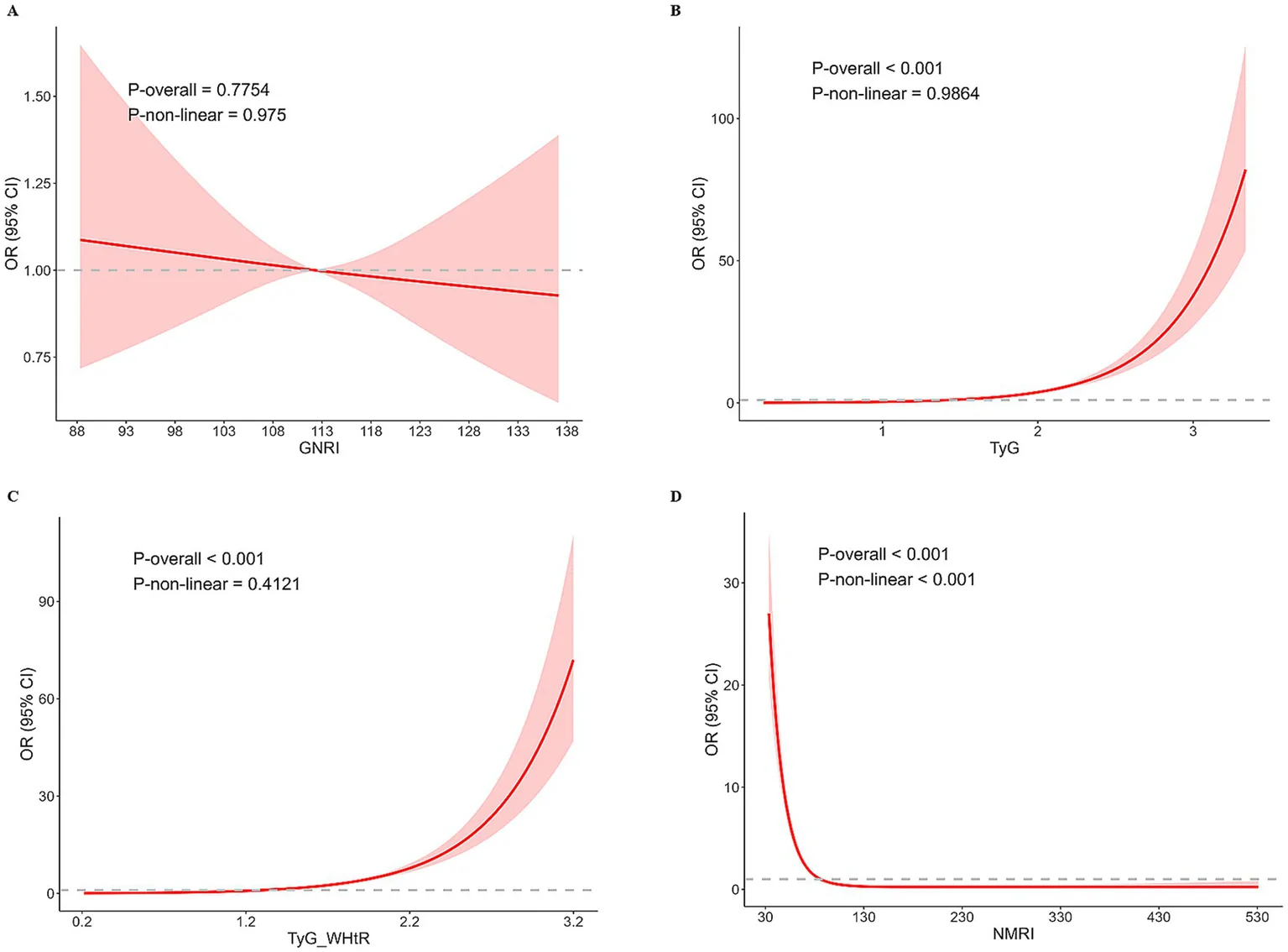
RCS analysis of the associations between GNRI, TyG, TyG-WHtR, NMRI and diabetes risk. **(A)** GNRI, **(B)** TyG, **(C)** TyG-WHtR, and **(D)** NMRI in relation to diabetes. The solid central line represents the adjusted hazard ratios (HRs), and the red shaded area represents the 95% confidence interval (CI). Inflection points are exploratory and should not be interpreted as definitive clinical thresholds. Model 3 adjustment was applied.

Figure 2 presents RCS analyses. GNRI exhibited no significant association with T2DM risk, while TyG and TyG-WHtR were significantly and positively associated with T2DM risk in a linear manner (*p* for overall < 0.001 for both). NMRI exhibited a significant non-linear inverse association with T2DM risk (*p* for overall < 0.001; *p* for non-linearity < 0.001), with risk appearing to decrease more steeply at lower NMRI levels, though this threshold is exploratory and requires prospective validation, and plateauing at higher levels.

### 3.5 Mediation and moderation analyses

**Figure 3:**
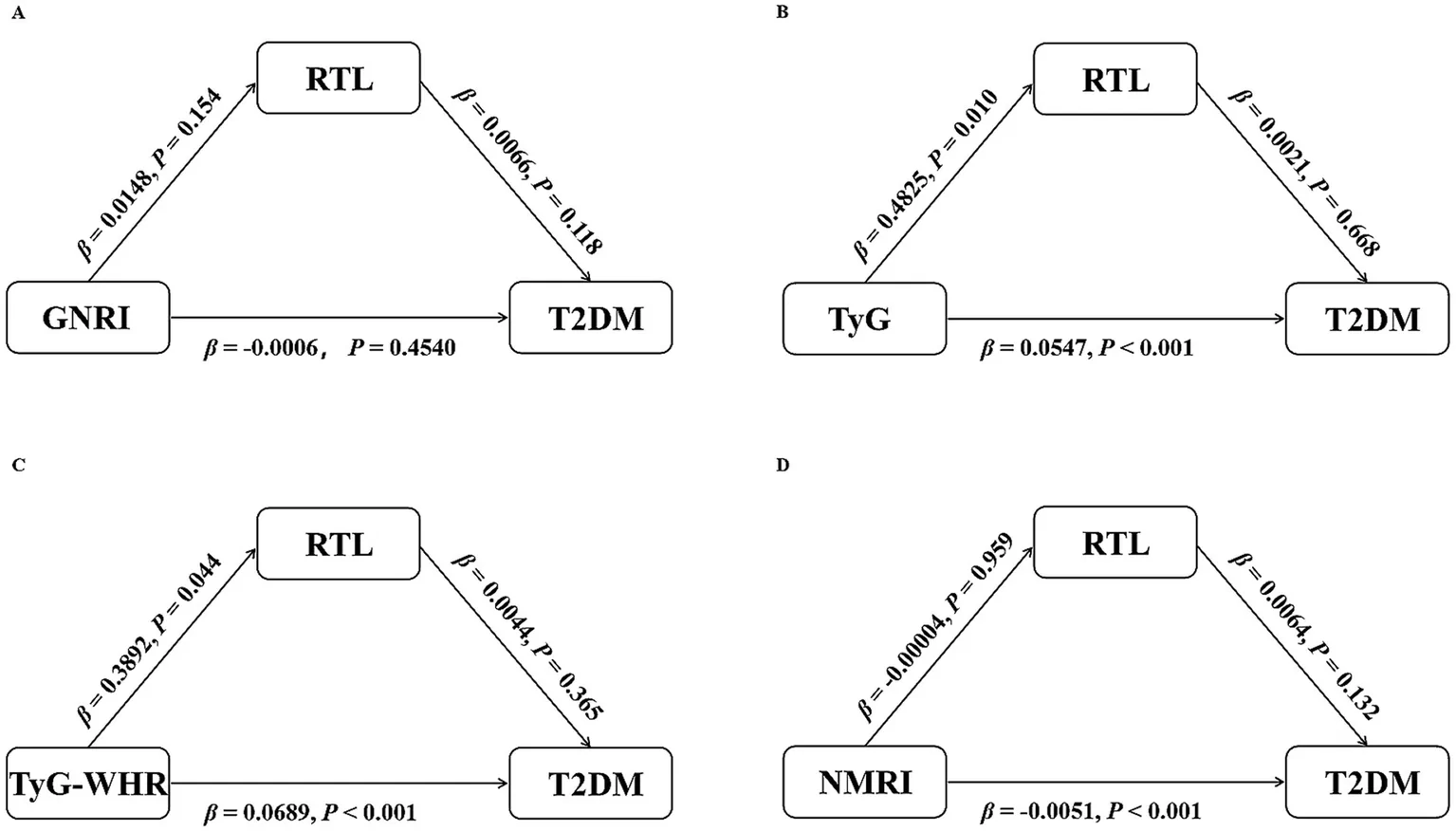
Relationship between GNRI, TyG, TyG-WHtR, NMRI, and diabetes. **(A)** GNRI → RTL → Diabetes, **(B)** TyG → RTL → Diabetes, **(C)** TyG-WHtR → RTL → Diabetes, and **(D)** NMRI → RTL → Diabetes. All models were adjusted for covariates as in Model 3.

Figure 3 and Supplementary Figure 2 presents that RTL did not significantly mediate the relationship between GNRI, TyG, TyG-WHtR, or NMRI and T2DM risk. The effects of GNRI on RTL and RTL on T2DM were not significant (both *p* > 0.05). TyG and TyG-WHtR were strongly associated with T2DM (both *p* < 0.001); however, their indirect effects through RTL were not significant. NMRI was inversely associated with T2DM but not significantly associated with RTL.

We explored whether RTL influenced the non-linear NMRI–T2DM risk association identified in the restricted cubic spline (RCS) analysis. Table 2 and Supplementary Figure 3 present the results of the moderation analysis. NMRI remained significantly inversely associated with T2DM (OR = 0.961; 95% CI: 0.956–0.966; *p* < 0.001) in the interaction model. The NMRI and RTL group interaction (long versus short) was significant (OR = 1.007; 95% CI: 1.000–1.014; *p* = 0.041). While statistically significant, the magnitude of this interaction is modest and its clinical significance requires further investigation. T2DM risk decreased more sharply with higher NMRI in the short RTL group, while the inverse association was less pronounced in the long RTL group (Supplementary Figure 3).

**Table 2:** Analysis of the moderating effects of RTL on the associations between NMRI and diabetes.

Characteristic	β	SE	*z*	*p*-value	OR (95% CI)
NMRI	−0.0399	0.0027	−14.810	<0.001	0.961 (0.956–0.966)
RTL (long vs. short)	−0.2365	0.2538	−0.9320	0.3510	0.789 (0.480–1.298)
NMRI × RTL	0.0069	0.0034	2.0435	0.0410	1.007 (1.000–1.014)

OR, Odds Ratio; CI, Confidence Interval. Model adjusted for Age, Gender, Education, Marriage, Occupation, Tea consumption status, Alcohol drinking status, Smoking status, Exercise level, History of hypertension, History of CVD, History of stroke, Family history of diabetes, Family history of CVD, Family history of stroke, HCY, TC, HDL-C, LDL-C, SCr.

### 3.6 Subgroup analyses

Supplementary Figure 4 summarizes the subgroup analyses. TyG and TyG-WHtR were positively associated with T2DM, with stronger links in hypertensive and nonagricultural workers. In contrast, NMRI demonstrated a consistent protective role across all subgroups, with stronger inverse effects observed in non-tea drinkers and inactive individuals, partly moderated by hypertension. GNRI’s association was significantly modified by occupation and lifestyle.

## 4 Discussion

This study systematically evaluated the association between four nutritional metabolic indices and T2DM in a large cohort. These results revealed that the TyG index and TyG-WHtR were positively associated with T2DM risk in a linear, dose-dependent manner. NMRI, a new composite index, was independently and inversely associated with T2DM, presenting a significant non-linear association, with stronger inverse associations observed among participants with shorter telomeres. Although RTL did not mediate the associations between the indices and T2DM, it significantly modified the effect, modifying the inverse association between NMRI and T2DM risk.

### 4.1 Interpretation of associations between Core metabolic markers and T2DM

These findings strongly support the use of TyG and TyG-WHtR as useful indicators of T2DM risk (4, 5, 24, 25), consistent with previous studies. The predictive power of TyG-WHtR is similar to, but not greater than, the original TyG index. These findings suggest that the primary predictive value lies in the metabolic dysregulation indicated by fasting TG and glucose, which reflects lipid–glucose interaction and IR. TyG-WHtR may not add significant predictive value, or its effects may already be included in the TyG index.

Unlike TyG-based indices, GNRI, which mainly reflects nutritional status, exhibited no significant associations with T2DM in this study. This may be due to our cohort of relatively young, rural adults, in whom severe protein-energy malnutrition is relatively rare (26, 27). GNRI may be less effective in identifying metabolic risk in younger, nonclinical populations. Additionally, the relationship between nutrition and diabetes is complex and possibly U-shaped. Although malnutrition is associated with metabolic risk through sarcopenia and inflammation (28), overnutrition drives obesity and IR (29). GNRI, primarily developed to assess nutritional risk, may not adequately capture this complex relationship well, especially in distinguishing overnutrition-related metabolic issues.

This study uniquely identified NMRI as being independently inversely associated with T2DM, presenting a non-linear dose–response relationship. The inverse association of NMRI may arise from its dual assessment of nutritional reserve and metabolic state. The GNRI component indicates good protein nutrition and body weight, supporting lean mass, reducing inflammation, and enhancing insulin sensitivity (30, 31). A higher NMRI score was also correlated with a lower TyG-WHtR, indicating better glycemic and lipid profiles and reduced central adiposity, leading to improved insulin sensitivity (32). Accordingly, NMRI may reflect the combined influence of low nutritional and metabolic risk. NMRI may be a useful indicator in research settings. However, before clinical implementation, it requires validation in prospective cohorts and assessment of its incremental predictive value beyond established models such as the Finnish Diabetes Risk Score (FINDRISC).

### 4.2 Complex role of RTL: moderation, not mediation

Path analysis revealed no significant mediating effect of RTL on the link between core exposure indices and T2DM, suggesting that these indices may not be associated with T2DM risk through telomere shortening pathways. Instead, RTL is a marker of biological aging or a comorbid state. This view aligns with the understanding that telomeres play a complex role in the pathogenesis of metabolic diseases. Some studies have found associations between leukocyte RTL and lipid parameters, but not with the severity of T2DM complications, indicating that telomere shortening may be a general aging biomarker rather than a direct cause of complications (33). Moreover, obesity and T2DM, which often occur together, are linked to shorter telomeres; however, oxidative stress and chronic inflammation may represent potential pathways underlying this association that warrant further investigation (34). This evidence suggests that RTL may primarily reflect an individual’s overall biological aging, independent of specific metabolic issues. The underlying pathways discussed above are putative and need to be verified by further mechanistic studies. These findings do not support a substantial mediating role for the metabolic nutritional indices studied in harmful effects.

The moderation analysis suggested a potential modification effect of RTL on the association between NMRI and T2DM, though the effect size was modest and its biological and clinical relevance remains uncertain. The inverse association of NMRI was stronger in those with shorter telomeres, indicating a nominal effect modification. Short telomeres indicate cellular aging and stress, increasing susceptibility to metabolic dysfunction (35, 36). Thus, individuals with shorter telomeres who maintain good metabolic health (higher NMRI) may experience a stronger inverse association with T2DM risk than those with longer telomeres. These findings highlight the importance of managing metabolic health in individuals experiencing accelerated biological aging.

Stratified analysis revealed stronger positive associations of TyG and TyG-WHtR with T2DM in the long RTL group. This counterintuitive finding could potentially be attributed to longer telomeres, which are associated with greater cellular repair capacity (37, 38). Under high metabolic stress, this may result in increased low-grade inflammation or represent compensatory metabolic adaptations (37, 38). This study suggests that mechanisms underlying high metabolic risk may vary among individuals with different aging profiles.

### 4.3 Subgroup analysis interpretation

Subgroup analyses revealed stronger associations of TyG and TyG-WHtR with T2DM in hypertensive individuals and nonagricultural workers. NMRI showed consistent protective effects across all subgroups, with stronger inverse associations observed in non-tea drinkers, non-drinkers, physically inactive individuals, and hypertensive patients. GNRI’s association was significantly modified by occupation, tea drinking, and hypertension status, suggesting that lifestyle and clinical factors may influence the relationship between nutritional status and diabetes risk.

### 4.4 Strengths and limitations

The strengths of this study included its large sample size, simultaneous comparison of novel and established indices, first exploration of the NMRI–T2DM association and RTL’s moderating role, and advanced statistical methods.

Several limitations should be acknowledged. First, the cross-sectional design precludes causal inference. Residual confounding may persist despite multivariable adjustment. Factors known to influence both telomere length and diabetes risk, including dietary patterns, specific medication details, inflammatory biomarkers, and per capita socioeconomic status, were not fully assessed. Serum creatinine was added as an adjustment covariate, and sensitivity analyses confirmed the robustness of the main findings. RTL measurements also carry inherent technical and biological variability. While standardized protocols and duplicate assays were used to minimize technical variation, biological variability driven by leukocyte composition remains. Finally, the geographically restricted cohort limits the generalizability of findings to other populations.

Future prospective studies are warranted to confirm causality, explore the interplay between NMRI and cellular senescence, and evaluate these indices for clinical diabetes risk stratification.

## 5 Conclusion

In summary, in this study cohort, TyG and TyG-WHtR indices were associated with increased odds of T2DM. Furthermore, NMRI, a newly developed composite measure encompassing both nutritional and metabolic health parameters, exhibited a significant non-linear inverse association with T2DM. This association was more pronounced in individuals with shorter telomeres. These findings indicate that biological aging may modulate the relationship between nutritional-metabolic status and diabetes risk, though causality cannot be inferred. Our results highlight the importance of integrating nutritional indices with aging biomarkers for personalized diabetes prevention, and future studies are warranted to validate these findings and explore the underlying mechanisms.

## Data Availability

The raw data supporting the conclusions of this article will be made available by the authors, without undue reservation.

## Supplementary material

SUPPLEMENTARY FIGURE 1 ROC curves of GNRI, TyG, TyG-WHtR, and NMRI for predicting diabetes in fully adjusted models. The models were adjusted for covariates as in Model 3.

SUPPLEMENTARY FIGURE 2 Mediation analysis showing the direct and indirect effects of various metabolic indices on diabetes risk, mediated by RTL. **(A)** GNRI → RTL → Diabetes, **(B)** TyG → RTL → Diabetes, **(C)** TyG-WHR → RTL → Diabetes, and **(D)** NMRI → RTL → Diabetes. ACME, Average Causal Mediation Effect; ADE, Average Direct Effect. All models were adjusted for covariates as in Model 3.

SUPPLEMENTARY FIGURE 3 Association between NMRI and predicted probability of diabetes, stratified by RTL. The models were adjusted for covariates as in Model 3.

SUPPLEMENTARY FIGURE 4 Forest plots showing the association of GNRI, TyG, TyG-WHtR, NMRI with diabetes risk across population subgroups. **(A)** GNRI, **(B)** TyG, **(C)** TyG-WHtR, and **(D)** NMRI in relation to diabetes. All models were adjusted for covariates as in Model 3, except for the stratified variable itself.
