# Supplementary material for: The novel nutritional metabolic risk index and established triglyceride-glucose indices in type 2 diabetes: modifying role of biological aging assessed by telomere length
Source: Front Nutr. 2026 Aug 12;13:1911472. doi: 10.3389/fnut.2026.1911472 (PMC13506887; doi:10.3389/fnut.2026.1911472)
Supplement: Supplementary file 1 [file Table_1.DOCX]

**Supplementary Table 1.** Baseline characteristics of participants according to T2DM status

| **Characteristic** | | **Total population N = 4,572** | **Normal**  **N = 3,557** | **T2DM**  **N = 1,015** | **P-value** |
| --- | --- | --- | --- | --- | --- |
| **n(%)** | |  |  |  |  |
| Gender | |  |  |  | 0.500 |
|  | Male | 1,889 (41.32%) | 1,460 (41.05%) | 429 (42.27%) |  |
|  | Female | 2,683 (58.68%) | 2,097 (58.95%) | 586 (57.73%) |  |
| Education | |  |  |  | 0.005 |
|  | ≤ Elementary School | 3,122 (68.29%) | 2,392 (67.25%) | 730 (71.92%) |  |
|  | ≥ Middle School | 1,450 (31.71%) | 1,165 (32.75%) | 285 (28.08%) |  |
| Marriage | |  |  |  | <0.001 |
|  | Married | 4,209 (92.06%) | 3,303 (92.86%) | 906 (89.26%) |  |
|  | Widowed/Separated/Divorced | 349 (7.63%) | 244 (6.86%) | 105 (10.34%) |  |
|  | Never Married | 14 (0.31%) | 10 (0.28%) | 4 (0.39%) |  |
| Occupation | |  |  |  | 0.300 |
|  | Farmer | 3,087 (67.52%) | 2,416 (67.92%) | 671 (66.11%) |  |
|  | Other | 1,485 (32.48%) | 1,141 (32.08%) | 344 (33.89%) |  |
| Tea consumption status | |  |  |  | <0.001 |
|  | Yes | 1,743 (38.12%) | 1,410 (39.64%) | 333 (32.81%) |  |
| Alcohol drinking status | |  |  |  | 0.200 |
|  | Yes | 307 (6.71%) | 248 (6.97%) | 59 (5.81%) |  |
| Smoking status | |  |  |  | 0.800 |
|  | Yes | 507 (11.09%) | 397 (11.16%) | 110 (10.84%) |  |
| Exercise level | |  |  |  | <0.001 |
|  | Unclassified | 520 (11.37%) | 417 (11.72%) | 103 (10.15%) |  |
|  | Insufficient | 3,143 (68.74%) | 2,479 (69.69%) | 664 (65.42%) |  |
|  | Sufficient | 284 (6.21%) | 203 (5.71%) | 81 (7.98%) |  |
|  | High | 625 (13.67%) | 458 (12.88%) | 167 (16.45%) |  |
| History of hypertension | |  |  |  | <0.001 |
|  | Yes | 2,401 (52.52%) | 1,738 (48.86%) | 663 (65.32%) |  |
| History of CVD | |  |  |  | <0.001 |
|  | Yes | 330 (7.22%) | 224 (6.30%) | 106 (10.44%) |  |
| History of stroke | |  |  |  | <0.001 |
|  | Yes | 88 (1.92%) | 54 (1.52%) | 34 (3.35%) |  |
| Family history of diabetes | |  |  |  | <0.001 |
|  | Yes | 303 (6.63%) | 195 (5.48%) | 108 (10.64%) |  |
| Family history of CVD | |  |  |  | 0.070 |
|  | Yes | 395 (8.64%) | 293 (8.24%) | 102 (10.05%) |  |
| Family history of stroke | |  |  |  | 0.021 |
|  | Yes | 249 (5.45%) | 179 (5.03%) | 70 (6.90%) |  |
| $\bar{\boldsymbol{X}}$**±S** | |  |  |  |  |
| Age (years) | | 57.00(50.00,66.00) | 56.00(49.00,65.00) | 62.00(53.00,67.00) | <0.001 |
| Height(cm) | | 160.00(154.50,165.50) | 160.00(155.00,165.00) | 160.00(154.00,166.00) | >0.900 |
| Weight(kg) | | 63.50(56.95,70.70) | 62.90(56.40,70.00) | 65.00(58.90,73.00) | <0.001 |
| BMI | | 24.90(22.70,27.20) | 24.70(22.50,27.00) | 25.70(23.30,28.00) | <0.001 |
| WC(cm) | | 86.90(80.50,93.30) | 86.10(80.00,92.70) | 88.80(82.70,95.60) | <0.001 |
| HC(cm) | | 94.30(90.90,98.00) | 94.00(90.70,97.60) | 95.30(91.90,99.10) | <0.001 |
| HCY(μmol/L) | | 16.00(12.70,21.90) | 16.10(12.70,21.80) | 15.80(12.80,22.10) | >0.9 |
| FBG (mmol/L) | | 5.49(5.01,6.31) | 5.30(4.91,5.70) | 8.07(7.26,10.41) | <0.001 |
| FINS (µIU/mL) | | 6.49(4.62,9.28) | 6.30(4.52,8.89) | 7.21(5.02,10.76) | <0.001 |
| TC (mmol/L) | | 4.84(4.21,5.44) | 4.79(4.19,5.36) | 5.02(4.28,5.76) | <0.001 |
| TG (mmol/L) | | 1.45(1.03,2.09) | 1.38(0.99,1.97) | 1.75(1.26,2.54) | <0.001 |
| HDL-C (mmol/L) | | 1.32(1.12,1.55) | 1.33(1.14,1.55) | 1.29(1.09,1.53) | 0.002 |
| LDL-C (mmol/L) | | 2.78(2.29,3.31) | 2.75(2.28,3.26) | 2.94(2.37,3.52) | <0.001 |
| Albumin (g/L) | | 47.70(44.20,51.70) | 47.60(44.10,51.60) | 47.90(44.20,52.10) | 0.200 |
| SCr (μmol/L) | | 80.00(69.84,92.00) | 79.43(69.70,91.33) | 81.47(70.17,93.59) | 0.030 |
| RTL | | 0.02(0.00,0.08) | 0.02(0.00,0.08) | 0.02(0.00,0.09) | 0.003 |
| GNRI | | 112.26(106.77,118.23) | 112.07(106.76,118.09) | 112.75(107.07,118.98) | 0.044 |
| TyG | | 1.43(1.04,1.88) | 1.28(0.95,1.67) | 2.05(1.63,2.52) | <0.001 |
| TyG-WHtR | | 1.31(0.93,1.77) | 1.18(0.86,1.55) | 1.94(1.51,2.37) | <0.001 |
| NMRI | | 86.00(63.43,120.80) | 95.11(72.56,132.45) | 58.35(46.99,75.31) | <0.001 |
| Values are presented as the mean ± SD or number (%). P-value obtained in the ANVOA or chi-squared test.  T2DM, type 2 diabetes mellitus; CVD, cardiovascular disease; BMI, body mass index; WC, waist circumstance; HC, hip circumstance; SBP, systolic blood pressure; DBP, diastolic blood pressure; TC, total cholesterol; TG, triglyceride; HDL-C, high-density lipoprotein cholesterol; LDL-C, low-density lipoprotein cholesterol; FPG, fasting blood-glucose; FINS, fasting insulin; SCr, Serum creatinine; RTL, relative leukocyte telomere length; GNRI, geriatric nutritional risk index; TyG, triglyceride-glucose index; TyG-WHtR, triglyceride-glucose index to waist-to-height ratio; NMRI, novel metabolic risk index. | | | | | |

**Supplementary Table 2.**  Predictive performance of four metabolic indices for diabetes risk

| **Index** | **AUC (95% CI)** | ***P*-value vs NMRI** | **NRI (95% CI)** | **IDI (95% CI)** |
| --- | --- | --- | --- | --- |
| Model 3 | 0.812 (0.797-0.827) | - | - | - |
| **GNRI** | 0.667 (0.649-0.667) | <0.001 | 0.004 (-0.006-0.014) | 0.0002 (-0.0001-0.0006) |
| **TyG** | 0.841 (0.828-0.841) | <0.001 | 0.866 (0.814-0.919) | 0.236 (0.220-0.252) |
| **TyG-WHtR** | 0.837 (0.824-0.837) | <0.001 | 0.847 (0.794-0.899) | 0.227 (0.212-0.243) |
| **NMRI** | 0.825 (0.810-0.825) | Ref | 0.781 (0.733-0.830) | 0.167 (0.157-0.178) |

AUC, Area Under the Curve; CI, Confidence Interval; NRI, Net Reclassification Improvement; IDI, Integrated Discrimination Improvement.Model 3: adjusted for Age, Gender, Education, Marriage, Occupation, Tea consumption status, Alcohol drinking status, Smoking status, Exercise level, History of hypertension, History of CVD, History of stroke, Family history of diabetes, Family history of CVD, Family history of stroke, HCY, TC, HDL-C, LDL-C, SCr.

**Supplementary Table 3.**  Pairwise comparison of AUC values using DeLong test

| **Comparison** | **AUC Difference (95% CI)** | ***P*-value** |
| --- | --- | --- |
| **NMRI vs GNRI** | 0.157 (0.134, 0.180) | <0.001 |
| **NMRI vs TyG** | -0.017 (-0.024, -0.010) | <0.001 |
| **NMRI vs TyG-WHtR** | -0.013 (-0.020, -0.006) | <0.001 |
| **TyG vs TyG-WHtR** | 0.004 (0.001, 0.007) | 0.003 |

AUC, Area Under the Curve; CI, Confidence Interval.AUC difference = AUC (first index) - AUC (second index). Model 3: adjusted for Age, Gender, Education, Marriage, Occupation, Tea consumption status, Alcohol drinking status, Smoking status, Exercise level, History of hypertension, History of CVD, History of stroke, Family history of diabetes, Family history of CVD, Family history of stroke, HCY, TC, HDL-C, LDL-C, SCr.

**Supplementary Table 4.** Associations between GNRI, TyG, TyG-WHtR, NMRI and T2DM based on weighted logistic regression analysis in the short RTL subgroup.

| **Characteristic** | | **Model 1** | | | | **Model 2** | | | | **Model 3** | | |
| --- | --- | --- | --- | --- | --- | --- | --- | --- | --- | --- | --- | --- |
|  |  | **OR** | **95% CI** | ***P*-value** |  | **OR** | **95% CI** | ***P*-value** |  | **OR** | **95% CI** | ***P*-value** |
| **GNRI** |  | 1.00 | 0.99, 1.01 | 0.800 |  | 1.00 | 0.99, 1.01 | 0.800 |  | 0.99 | 0.98, 1.01 | 0.300 |
| Quartile | Q1 | Ref |  |  |  | Ref |  |  |  | Ref |  |  |
|  | Q2 | 0.97 | 0.72, 1.29 | 0.800 |  | 0.94 | 0.70, 1.26 | 0.700 |  | 0.85 | 0.63, 1.15 | 0.300 |
|  | Q3 | 1.14 | 0.86, 1.51 | 0.400 |  | 1.07 | 0.80, 1.42 | 0.700 |  | 0.95 | 0.71, 1.28 | 0.800 |
|  | Q4 | 0.99 | 0.73, 1.33 | >0.900 |  | 0.92 | 0.68, 1.25 | 0.600 |  | 0.82 | 0.60, 1.13 | 0.200 |
| **TyG** |  | 7.88 | 6.41, 9.78 | <0.001 |  | 7.9 | 6.41, 9.82 | <0.001 |  | 8.73 | 6.96, 11.0 | <0.001 |
| Quartile | Q1 | Ref |  |  |  | Ref |  |  |  | Ref |  |  |
|  | Q2 | 2.87 | 1.66, 5.20 | <0.001 |  | 2.81 | 1.62, 5.10 | <0.001 |  | 2.96 | 1.69, 5.43 | <0.001 |
|  | Q3 | 6.98 | 4.24, 12.20 | <0.001 |  | 6.87 | 4.16, 12.00 | <0.001 |  | 7.29 | 4.36, 12.9 | <0.001 |
|  | Q4 | 30.70 | 19.00, 52.90 | <0.001 |  | 30.2 | 18.70, 52.20 | <0.001 |  | 33.5 | 20.2, 59.5 | <0.001 |
| **TyG_WHtR** |  | 7.79 | 6.33, 9.66 | <0.001 |  | 7.73 | 6.28, 9.60 | <0.001 |  | 8.65 | 6.88, 11.0 | <0.001 |
| Quartile | Q1 | Ref |  |  |  | Ref |  |  |  | Ref |  |  |
|  | Q2 | 3.51 | 1.96, 6.68 | <0.001 |  | 3.41 | 1.91, 6.50 | <0.001 |  | 3.52 | 1.95, 6.79 | <0.001 |
|  | Q3 | 8.98 | 5.25, 16.60 | <0.001 |  | 8.90 | 5.20, 16.40 | <0.001 |  | 9.31 | 5.37, 17.4 | <0.001 |
|  | Q4 | 34.80 | 20.70, 63.40 | <0.001 |  | 34.10 | 20.30, 62.20 | <0.001 |  | 37.1 | 21.5, 69.4 | <0.001 |
| **NMRI** |  | 0.96 | 0.96, 0.97 | <0.001 |  | 0.96 | 0.96, 0.97 | <0.001 |  | 0.96 | 0.96, 0.97 | <0.001 |
| Quartile | Q1 | Ref |  |  |  | Ref |  |  |  | Ref |  |  |
|  | Q2 | 0.25 | 0.19, 0.33 | <0.001 |  | 0.25 | 0.19, 0.33 | <0.001 |  | 0.25 | 0.19, 0.32 | <0.001 |
|  | Q3 | 0.09 | 0.07, 0.13 | <0.001 |  | 0.09 | 0.07, 0.13 | <0.001 |  | 0.09 | 0.06, 0.13 | <0.001 |
|  | Q4 | 0.04 | 0.02, 0.06 | <0.001 |  | 0.04 | 0.02, 0.06 | <0.001 |  | 0.04 | 0.02, 0.06 | <0.001 |
| OR, Odds Ratio; CI, Confidence Interval. Model 1: unadjusted; Model 2: adjusted for Age, Gender; Model 3: adjusted for Age, Gender, Education, Marriage, Occupation, Tea consumption status, Alcohol drinking status, Smoking status, Exercise level, History of hypertension, History of CVD, History of stroke, Family history of diabetes, Family history of CVD, Family history of stroke, HCY, TC, HDL-C, LDL-C, SCr. | | | | | | | | | | | | |

**Supplementary Table 5.** Associations between GNRI, TyG, TyG-WHtR, NMRI and T2DM based on weighted logistic regression analysis in the long RTL subgroup.

| **Characteristic** | | **Model 1** | | | | **Model 2** | | | | **Model 3** | | |
| --- | --- | --- | --- | --- | --- | --- | --- | --- | --- | --- | --- | --- |
|  |  | **OR** | **95% CI** | ***P*-value** |  | **OR** | **95% CI** | ***P*-value** |  | **OR** | **95% CI** | ***P*-value** |
| **GNRI** |  | 1.01 | 1.00, 1.02 | 0.069 |  | 1.00 | 0.99, 1.01 | 0.800 |  | 1.00 | 0.99, 1.01 | 0.700 |
| Quartile | Q1 | Ref |  |  |  | Ref |  |  |  | Ref |  |  |
|  | Q2 | 1.04 | 0.79, 1.38 | 0.800 |  | 0.99 | 0.75, 1.32 | >0.900 |  | 0.95 | 0.71, 1.28 | 0.700 |
|  | Q3 | 1.08 | 0.81, 1.43 | 0.600 |  | 0.97 | 0.73, 1.29 | 0.800 |  | 0.91 | 0.68, 1.23 | 0.600 |
|  | Q4 | 1.36 | 1.04, 1.77 | 0.025 |  | 1.12 | 0.85, 1.48 | 0.400 |  | 1.03 | 0.77, 1.37 | 0.900 |
| **TyG** |  | 9.12 | 7.41, 11.30 | <0.001 |  | 9.93 | 8.01, 12.40 | <0.001 |  | 13.10 | 10.20, 16.90 | <0.001 |
| Quartile | Q1 | Ref |  |  |  | Ref |  |  |  | Ref |  |  |
|  | Q2 | 2.77 | 1.77, 4.46 | <0.001 |  | 2.81 | 1.79, 4.54 | <0.001 |  | 2.84 | 1.78, 4.63 | <0.001 |
|  | Q3 | 6.67 | 4.40, 10.50 | <0.001 |  | 6.91 | 4.53, 10.90 | <0.001 |  | 8.28 | 5.31, 13.30 | <0.001 |
|  | Q4 | 29.30 | 19.60, 45.60 | <0.001 |  | 32.7 | 21.60, 51.20 | <0.001 |  | 41.60 | 26.60, 67.30 | <0.001 |
| **TyG_WHtR** |  | 9.45 | 7.64, 11.80 | <0.001 |  | 10.40 | 8.35, 13.10 | <0.001 |  | 13.80 | 10.70, 18.00 | <0.001 |
| Quartile | Q1 | Ref |  |  |  | Ref |  |  |  | Ref |  |  |
|  | Q2 | 2.45 | 1.57, 3.92 | <0.001 |  | 2.48 | 1.58, 3.97 | <0.001 |  | 2.52 | 1.59, 4.07 | <0.001 |
|  | Q3 | 6.70 | 4.48, 10.40 | <0.001 |  | 6.87 | 4.56, 10.70 | <0.001 |  | 8.18 | 5.33, 13.00 | <0.001 |
|  | Q4 | 27.70 | 18.70, 42.60 | <0.001 |  | 30.90 | 20.70, 47.90 | <0.001 |  | 39.90 | 25.70, 63.90 | <0.001 |
| **NMRI** |  | 0.97 | 0.97, 0.97 | <0.001 |  | 0.97 | 0.96, 0.97 | <0.001 |  | 0.97 | 0.96, 0.97 | <0.001 |
| Quartile | Q1 | Ref |  |  |  | Ref |  |  |  | Ref |  |  |
|  | Q2 | 0.24 | 0.19, 0.31 | <0.001 |  | 0.22 | 0.17, 0.28 | <0.001 |  | 0.20 | 0.15, 0.26 | <0.001 |
|  | Q3 | 0.10 | 0.07, 0.13 | <0.001 |  | 0.09 | 0.06, 0.12 | <0.001 |  | 0.07 | 0.05, 0.10 | <0.001 |
|  | Q4 | 0.04 | 0.02, 0.05 | <0.001 |  | 0.03 | 0.02, 0.05 | <0.001 |  | 0.02 | 0.01, 0.04 | <0.001 |

OR, Odds Ratio; CI, Confidence Interval. Model 1: unadjusted; Model 2: adjusted for Age, Gender; Model 3: adjusted for Age, Gender, Education, Marriage, Occupation, Tea consumption status, Alcohol drinking status, Smoking status, Exercise level, History of hypertension, History of CVD, History of stroke, Family history of diabetes, Family history of CVD, Family history of stroke, HCY, TC, HDL-C, LDL-C, SCr.
